# West End Walkers 65+: A randomised controlled trial of a primary care-based walking intervention for older adults: Study rationale and design

**DOI:** 10.1186/1471-2458-11-120

**Published:** 2011-02-19

**Authors:** Freya MacMillan, Claire Fitzsimons, Karen Black, Malcolm H Granat, Margaret P Grant, Madeleine Grealy, Hazel Macdonald, Alex McConnachie, David A Rowe, Rebecca Shaw, Dawn A Skelton, Nanette Mutrie

**Affiliations:** 1 School of Psychological Sciences and Health, University of Strathclyde, Jordanhill Campus, 76 Southbrae Drive, Glasgow, G13 1PP, UK; 2 School of Health, Glasgow Caledonian University, Cowcaddens Road, Glasgow, G4 0BA, UK; 3 Robertson Centre for Biostatistics, University of Glasgow, University Avenue, Glasgow, G12 8QQ, UK; 4 Public Health and Health Policy, University of Glasgow, 1 Lilybank Gardens, Glasgow, G12 8RZ, UK

## Abstract

**Background:**

In Scotland, older adults are a key target group for physical activity intervention due to the large proportion who are inactive. The health benefits of an active lifestyle are well established but more research is required on the most effective interventions to increase activity in older adults. The 'West End Walkers 65+' randomised controlled trial aims to examine the feasibility of delivering a pedometer-based walking intervention to adults aged ≥65 years through a primary care setting and to determine the efficacy of this pilot. The study rationale, protocol and recruitment process are discussed in this paper.

**Methods/Design:**

The intervention consisted of a 12-week pedometer-based graduated walking programme and physical activity consultations. Participants were randomised into an immediate intervention group (immediate group) or a 12-week waiting list control group (delayed group) who then received the intervention. For the pilot element of this study, the primary outcome measure was pedometer step counts. Secondary outcome measures of sedentary time and physical activity (time spent lying/sitting, standing or walking; *activ*PAL™ monitor), mood (Positive and Negative Affect Schedule), functional ability (Perceived Motor-Efficacy Scale for Older Adults), quality of life (Short-Form (36) Health Survey version 2) and loneliness (UCLA Loneliness Scale) were assessed. Focus groups with participants and semi-structured interviews with the research team captured their experiences of the intervention. The feasibility component of this trial examined recruitment via primary care and retention of participants, appropriateness of the intervention for older adults and the delivery of the intervention by a practice nurse.

**Discussion:**

West End Walkers 65+ will determine the feasibility and pilot the efficacy of delivering a pedometer-based walking intervention through primary care to Scottish adults aged ≥65 years. The study will also examine the effect of the intervention on the well-being of participants and gain an insight into both participant and research team member experiences of the intervention.

**Trial registration number:**

ISRCTN: ISRCTN70658148

## Background

Physical activity has been referred to as a 'miracle cure' because of the potential health benefits that can accrue from being regularly active [1]. The current physical activity recommendation for health improvement and maintenance for older adults is to achieve 30 minutes of moderate intensity physical activity on most days of the week [2]. Throughout this paper the term 'older adult' will be used to refer to an individual aged ≥65 years. The proportion of adults meeting the physical activity guideline on at least five days of the week in Scotland declines with increasing age; 78% of men and 83% of women aged 65-74 years and 89% of men and 94% of women aged ≥75 years currently do not meet that recommendation [3]. The Scottish physical activity strategy "let's make Scotland more active" has a target of 50% of adults meeting the physical activity recommendation by 2022 [4]. Older adults are a key segment of the population for physical activity intervention if Scotland is to reach the 2022 target, because of the low proportion of older adults meeting the physical activity guideline [5] and an ageing population structure [6]. In addition, older adults may have the most to gain from physical activity intervention as a consequence of physical [7-9] and cognitive [10,11] functions deteriorating with age. Being regularly physically active has physiological (e.g. reduced risk of overall mortality, cardiovascular disease, obesity, Type 2 diabetes, osteoporosis and breast and colo-rectal cancer), psychological (e.g. reduced anxiety and depression risk and reduced risk or decreased rate of dementia and memory loss development), social (e.g. interaction) and overall well-being benefits (e.g. increased energy, vitality and improved mood and sleep pattern) [2,12]. Regular physical activity has a number of benefits that are particularly important for older adults. It can: a) slow the age-related decline in functional capacity; b) improve mobility and independence thus making activities of daily living easier; c) slow age-related declines in cognition or even improve cognitive functioning; d) reduce the risk of falling; and e) benefit areas of mental health such as social interaction and overall well-being [2,12-14].

Walking is an ideal mode of physical activity for older adults and has been described as 'the nearest activity to perfect exercise' [15] (p. 328) as it is a cheap and safe way of increasing physical activity participation with minimal adverse effects [15]. For previously sedentary adults, walking can result in physiological health benefits including decreases in body weight, body mass index, percentage body fat and resting diastolic blood pressure [16] as well as psychological health benefits such as positive effects on mood [17]. In a systematic review by Ogilvie et al. [18] of the evidence for successful walking interventions, the authors identified a lack of peer reviewed literature on how best to support older people. The authors also concluded that walking interventions tailored to the individual and delivered on a one-to-one, household or group basis are the most successful at encouraging increases in walking participation in those that are most sedentary and/or most motivated to change [18]. The review found evidence that pedometers are effective tools to increase walking participation in adults but there is a need for research to examine who can benefit the most from pedometer-based walking programmes and which elements of the interventions are most important to their success. In addition, the review called for studies to be conducted outside of the USA and Australia where most of the current literature was generated and also to focus on longer term effects.

There is growing interest around the world in the health implications of sedentary behaviour (time spent sitting and lying), independent of physical activity levels. A number of recent policy documents have highlighted concern about the high levels of sedentary behaviours in the Scottish population [4,19]. Recent evidence suggests a dose-response association between sitting times and mortality from all causes and cardiovascular disease, independent of leisure time physical activity [20]. If an individual achieves the recommended amount of physical activity, his/her health may still be at risk if sedentary for many hours. Recent research from Australia suggests that those who spend more time in sedentary behaviour but are sufficiently active (at least 2.5 hrs of activity/week) and those who are insufficiently active but spend less time in sedentary behaviours have a similar risk of being overweight or obese [21]. A study using the *activ*PAL™ to assess activity patterns in 20 older Scottish adults (mean age 74.0 ± 5.3 years) found that on average 18 hours/day were spent in sedentary behaviours [22].

Previous research conducted by our group found a 12-week individualised pedometer-based graduated walking programme ('Walking for Well-being in the West'(WWW)), delivered with a series of physical activity consultations, significantly increased walking behaviour, improved mood and decreased self reported sitting time in Scottish adults aged 18-65 years [17,23] over a 3 month period. The WWW intervention was based on the recommendations from Ogilvie and colleagues' review [18], consisting of a one-to-one, theory-driven, individualised programme. The consultation was based on established guidelines [24] and adapted for walking behaviour. Specific behaviour change techniques that were used included information provision on the link between walking and health, setting graded tasks (pedometer step counts), identifying barriers and ways to overcome them, prompting self-monitoring by use of the pedometer, identifying social support and relapse prevention. All of these techniques have been recognised as having evidence for behaviour change and are used as described by Abraham et al. [25]. The methods of the present study will determine if the same intervention that was used in WWW could be used to increase walking behaviour in adults aged ≥65 years who do not currently meet the physical activity recommendations.

WWW was based in a community setting but delivered by a research team. Little is known regarding the feasibility of delivering such walking interventions by professionals (not researchers) through primary care. Therefore this study was based in a primary care setting, specifically in a general practice (family practice) and delivered by a practice nurse. Recruitment was via a general practice in this study for two main reasons. Firstly, it is a convenient location to reach older adults, as older adults attend their general practice regularly and more often than younger individuals. General practitioner (family physician) consultation rates in Scotland increase from the 45-54 year age group upwards [26]. In Scotland, 97% of the population is registered with a general practitioner/health practitioner [13] and 84% of patients have visited their local general practice team at least once in 2008/2009 [27]. Secondly, the 'let's make Scotland more active' [4] strategy recommended that all patients coming into contact with primary care professionals should be offered counselling for physical activity tailored to individual needs. Thus there is a need to determine practical strategies that such professionals could use. A general practice setting was selected to facilitate future implementation via primary care. This study will explore the use of a non-physician delivery model by employing a practice nurse as the intervention deliverer. Given time constraints on physicians, the use of non-physician delivery models has been recognised as a significant research area [28]. Appropriately trained nurses have been shown to produce equally high quality care as primary care doctors and achieve equally as good health outcomes for patients [29].

In 2008 National Health Service (NHS) Health Scotland developed a resource ('Energising Lives' [13]) for primary care staff and other health professionals to 'provide guidance on how to offer routine advice and encouragement to patients around physical activity' (p. 5) by including information on the benefits of physical activity and the physical activity recommendations for health. Although there have been various types of engagement across primary care to increase physical activity participation, there is currently no consensus evidence as to what is the best approach to promoting physical activity via primary care settings. Previous physical activity intervention studies delivered via primary care were reviewed by the National Institute for Health and Clinical Excellence (NICE) [30] in 2006. Eleven studies were included in the review, with six studies reporting significant increases in physical activity. The interventions included brief verbal advice, referrals, and motivational interviews delivered by health promotion specialists or researchers. Two studies delivered brief verbal advice and significantly increased physical activity [31,32]. There are limitations with the evidence from these studies. Specifically, both studies lacked a control condition, were based in New Zealand and Australia rather than the UK, used subjective measures of activity, and used a general practitioner/family physician to deliver the intervention (a model unlikely to be adopted in the UK due to constraints on general practitioner time). Of eight UK based studies conducted in primary care since the publication of the NICE guidelines which offered exercise/physical activity support [33-40], only one of these specifically targeted older adults (women aged ≥70 years) [39]) and four included an objective assessment of physical activity [36-39] (although one study used self-reported step counts as opposed to monitoring stored counts [37]). Four of the eight studies incorporated a pedometer into their intervention [33,37-39]. The results suggested pedometer use can increase physical activity via primary care, and one study reported a 101% increase in step counts after 12 weeks [37]. From these primary care-based studies it can be summarised that there is a paucity of research conducted in the UK looking at physical activity interventions that: a) target older adults; b) measure physical activity objectively to assess the efficacy of the interventions; c) have a graduated pedometer-based walking programme based on the current physical activity recommendations; and d) do not use highly trained members of primary care or researchers to recruit participants, deliver the intervention and perform follow-up appointments.

The West End Walkers study (WEW65+) has been designed as an exploratory trial, as described in phase II of the Medical Research Council's framework for the evaluation of complex interventions [41] and as a feasibility and pilot study, as described by the National Institute for Health Research Evaluation [42]. A feasibility/pilot study not only tests (on a smaller level than a main study) the likely efficacy of the intervention (such as the anticipated effect size of the primary outcome) but also tests whether all elements of the planned study can be implemented in practice and work together [42]. Such pilot work, when combined with a feasibility study, also tests intervention characteristics such as anticipated levels of recruitment, how easily the intervention can be implemented by the planned delivery team and whether the intervention is appropriate (e.g. in terms of time commitment, technological and skills demands, and accessibility) for the target population. The design and appropriateness of the intervention and the research protocol and how they impact on these issues (e.g. delivery, recruitment, uptake, retention, calibration, ease of data collection, implications of data for subsequent analysis) are vital for the success of both a pilot study and any subsequent full trial. To ensure that this pilot study took account of prior evidence and theory about behaviour change, implementation failures, and engagement in research, a logic model was developed of the initial protocol and this model was used along with key criteria from the RE-AIM framework [43], to strengthen our research plans. A separate paper has been written to describe this process (Blamey A, MacMillan F, Evans A, Fitzsimons C, Mutrie N: Using programme theory to strengthen research protocol and intervention design: A randomised controlled trial of a walking intervention for older adults (West End Walkers 65+), submitted). These steps were taken to strengthen the robustness of the pilot, to enhance the study protocol for a potential larger trial and enhance the generalisability of learning for general practice from both of these research stages.

To summarise, this study was designed to fill several research gaps in the literature. This study will explore the efficacy of a walking intervention (already shown to successfully increase walking in young to middle-aged adults [17]) in a randomised controlled trial with an older adult population in the UK and using an objective measurement of physical activity. The efficacy of the intervention on areas of health important to older adults will also be assessed using specific questionnaires shown to be valid and reliable measures in adult groups. The appropriateness of the physical activity recommendations and the walking intervention for older adults will also be explored. In addition this study will examine the feasibility of delivering the walking intervention in a primary care setting (general practice) to provide a practical solution to increasing physical activity levels of older adults, facilitating future implementation via this setting. The study will also look at the feasibility of delivering the walking intervention by a practice nurse (a more likely delivery mode in the UK than via general practitioners who have greater time constraints). Older adults were specifically targeted in this study due to the low proportion of Scottish older adults that currently meet the physical activity recommendations [3]. Participants were recruited via a general practice as a means of accessing older adults, due to the high proportion of older adults that attend their general practice in Scotland [13].

### Aim

This paper provides details of the rationale and study design (including information on the outcome measures assessed and recruitment process). The WEW65+ study aims to examine the delivery of a pedometer-based graduated walking programme in combination with physical activity consultations, through a primary care setting (general practice), to older Scottish adults who are currently not meeting the physical activity recommendations. The feasibility issues are: recruitment of participants into the study (who is targeted versus who is actually recruited) and delivery of the intervention via a primary care setting (general practice); delivery of the intervention by a practice nurse; practicality of administering the outcome measures; and whether the intervention is acceptable and enjoyable for an older adult population in a real-life setting. Determining the efficacy of the intervention in an older adult population (≥65 years) is also a pilot element of this study as the intervention has previously been shown to successfully increase walking participation in adults aged 18-65 years [17] but has not been tested in older adults. For the pilot component of this study, efficacy of the intervention will be evaluated by looking at the effect of the intervention on step counts (primary outcome), activity patterns and sedentary behaviour, mood, quality of life, perceived motor-efficacy and loneliness (secondary outcomes). Another pilot element of this study will be to assess if all components of the study can be implemented simultaneously and work together to have an effect on physical activity before planning a full trial.

The main research questions are:-

• Is the recruitment and retention strategy successful?

• Is the intervention acceptable, appropriate, accessible and useable for this group?

• What is the efficacy of the intervention on outcome measures?

• Are the outcome measures acceptable for this group?

## Methods/Design

### Ethical Approval

All procedures were approved by the NHS Greater Glasgow and Clyde and University of Strathclyde Ethics Committees and were carried out in accordance with the Declaration of Helsinki. Written informed consent was obtained from all participants.

### Power Calculation

For the pilot element of this study, sample size was estimated based on the primary outcome measure, pedometer steps/day. Based on findings from previous work conducted by this research team a conservative estimate of the potential effect in this study was a mean increase of 2,000 steps/day [17]. From prior research [44,45], a conservative estimate of the standard deviation for steps/day in older adults is 3,000 steps. This corresponds to an effect size of *D *= .67 [46]. Additionally, pedometer counts from older adults have been shown to be highly reliable [44], thus a high correlation between baseline and post-intervention data can be expected. Using the Power and Precision software, sample size for a Repeated Measures ANOVA was calculated for *D *= .67, α = .05, an inter-trial correlation of *r *= .60, and a desired power of *β *= .80. This yielded a sample size of 16 participants per group. Assuming a 30% loss in follow-up, a sample size of 46 (23 per group) was agreed as the target.

### Recruitment process

Recruitment commenced in August 2009. Medical records were screened for contraindications to physical activity [47] by a general practitioner. Computer random sequence generation was used to determine the order of screening. Recruitment was via letter sent from a general practitioner. Individuals were asked to indicate on a reply slip whether they would be interested in taking part. Positive responders indicated their preferred method of contact (email, post or telephone). A researcher contacted those wishing to take part to assess current physical activity participation and to schedule the first appointment. An open-ended question for negative responders to provide a reason why they did not want to take part was included in the recruitment letter. Reminder letters were sent out at least two weeks after initial study invitations to individuals that did not respond to the initial recruitment letter. Negative responders who did not give a reason for declining participation in their first reply were sent a further letter asking for help in trying to understand why the study did not appeal to them to assist the research team with future study recruitment.

### Study population

Inclusion criteria were as follows: aged ≥65 years; able to understand the study rationale; living independently; English speaking and not meeting the current physical activity recommendations (defined as being in the pre-contemplation, contemplation or preparation stages of change from the Transtheoretical Model of behaviour change in relation to meeting the current physical activity recommendations [48] (see table 1 for descriptions)). After discussion with a researcher on the telephone, individuals who were already regularly physically active (e.g. in stage 4 or 5) were excluded from the study. Two participants recruited into the study were from the same household (the second member of the household was not invited into the study until the first member had completed participation in the study).

**Table 1:** Description of the stages of change in relation to meeting the current physical activity recommendations

Stage	Stage name	Description of individual in terms of physical activity participation
1	Pre-contemplation	Not regularly physically active and do not intend to be in the next 6 months
2	Contemplation	Not regularly physically active but are thinking about becoming more active in the next 6 months
3	Preparation	Do some physical activity but do not participate in regular physical activity
4	Action	Regularly active but for less than 6 months
5	Maintenance	Regularly active for over 6 months

### The intervention

Glasziou and colleagues [49] highlight the importance of fully describing intervention protocols in enough detail for others to replicate the intervention. The authors acknowledge that full reporting may not be feasible in scientific journals where space is limited and thus recommend providing links to websites where full details are available [49]. Intervention pro formas for this study are detailed in the intervention manual which can be accessed at http://www.strath.ac.uk/humanities/courses/physicalactivityforhealth/staff/mutrienanetteprof/ or by contacting the chief investigator. A 30 minute physical activity consultation was delivered individually to each participant by a practice nurse who had been trained in consultation procedures. To examine delivery of the intervention by a practice nurse and to assess consultation fidelity, audio recordings of consultations were made. NHS Health Scotland has provided physical activity consultation training which can be undertaken by anyone with an interest and basic knowledge in health and physical activity (i.e. college or university level knowledge is not required) and is delivered over 1-2 days. The lead trainer for these courses conducted a one-to-one training session with the study practice nurse over 1 day and was available for clarification and guidance as the nurse practiced these skills before delivering a consultation with participants. The consultations followed recommended guidelines [24], were based on the social cognitive model of behaviour change as a theoretical framework, and have previously been shown to successfully increase physical activity participation in adults [17,50,51]. Details of the behaviour change processes targeted in the consultations are provided in Table 2. The intervention involved two consultations. The aim of the initial consultation was to increase walking participation. A second consultation focussed on relapse prevention (12 weeks after the first consultation) and aimed to maintain walking behaviour.

**Table 2:** Description of the behaviour change techniques targeted in the consultations

Behaviour change technique	How the technique was used in the consultation
Motivation for taking part	Discussion of why participant interested in the project
Information on the link between walking and health	Reflection on role of physical activity for health and well being
Self reflection on pros and cons of increasing walking	Weighing up pros and cons of increasing walking
Graded tasks	Graded walking goals set tailored to each individuals baseline pedometer readings
Instruction	Using the pedometer
Self-monitoring	Using the pedometer to monitor progress towards goals
Identifying and overcoming barriers	Recognising barriers and inviting participant to consider ways to overcome
Relapse prevention	For immediate intervention group only at consultation two: discussion of avoiding lapses, recognising situations which make walking difficult, thinking of alternatives.

A 12-week individualised walking programme and pedometer were also given to participants. Details of the walking programme are given in Table 3. A cadence of 100 steps/minute has become a widely-accepted population guideline for an adult walking at a moderate pace, and has been confirmed in two recent controlled studies of overground walking [52,53]. Thus increasing step count by 3,000 steps/day equates to approximately 30 minutes of moderate intensity physical activity. It is important to note however that limited research has examined step rate specifically in older adults with only one of these studies including adults aged 50-65 years [53].

**Table 3:** Details of the 12-week pedometer based graduated walking programme

Week	Goal
Week 1-2	To increase the individual's average daily step count by 1500 steps above their baseline value on at least 3 days of the week
Week 3-4	To increase the individual's average daily step count by 1500 steps above their baseline value on at least 5 days of the week
Week 5-6	To increase the individual's average daily step count by 3000 steps above their baseline value on at least 3 days of the week
Week 7-8	To increase the individual's average daily step count by 3000 steps above their baseline value on at least 5 days of the week
Week 9-12	To maintain their walking level aiming for the week 7 goal

A local walking group was set up and led by a trained walk leader to help participants increase walking participation by providing peer support and a social support network. Feedback from participants in the WWW trial suggested the provision of a local walking group would have enhanced the study [23]. Paths for All (http://www.pathsforall.org.uk), a national charity which promotes walking for health, assisted in the establishment (including the training of walk leaders by completion of a one day course) and sustainability of the walking group. An attendance record was kept to determine how many participants used the group and how often.

### Design

There were two groups in the study; an immediate intervention group (immediate group) and a 12-week waiting list group (delayed group). The design of the study was a 12-week randomised controlled trial with a 12-week follow up for the intervention group. The immediate group received the intervention when they were entered into the study. The delayed group acted as a control group and were instructed to continue with their normal physical activity behaviour for the first 12 weeks of the study before receiving the same intervention as the immediate group.

### Procedures

**Figure 1:**
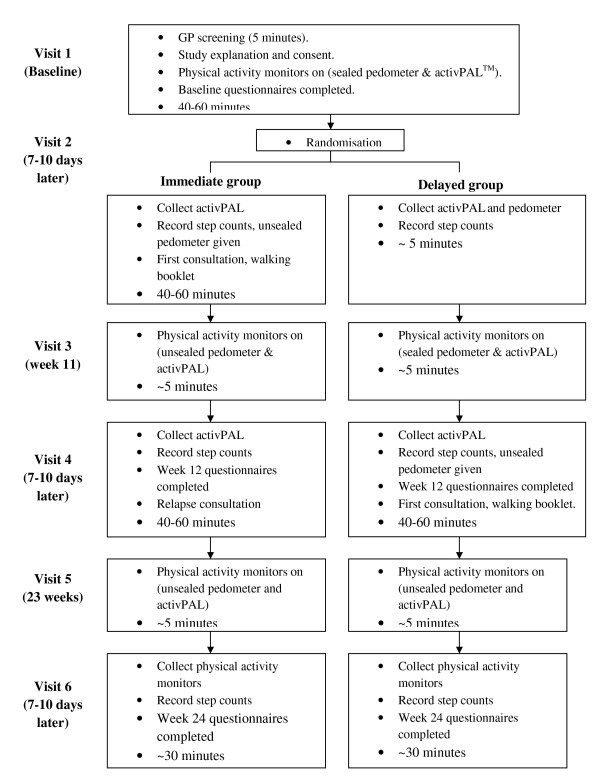
**Flowchart of participant visits through the study**.

Participants were invited to attend six study visits over 24 weeks (Figure 1). At visit 1, participants met with the practice nurse to go over the study protocol, discussed their present health with the general practitioner, completed informed consent and questionnaires and were fitted with a sealed pedometer (so that participants were unaware of their step counts before being given the intervention) and an *activ*PAL. All participants were instructed to continue with their normal physical activity patterns. At visit 2, one week after visit 1, individuals were randomised, on a one-to-one basis, into the immediate or delayed group by selecting a sealed envelope. Those who achieved ≥70,000 steps/week at baseline were classed as regularly physically active and excluded from participation as this equates to ≥150 minutes of moderate physical activity/week [54]. Participants in the delayed group were instructed at this visit to continue with their normal physical activity patterns for the next 12 weeks. At visit 3, 11 weeks after visit 1, all participants were fitted with an *activ*PAL and the immediate group continued to wear an unsealed pedometer (as they had had the intervention and were using the pedometer as part of the walking programme) whilst the delayed group were fitted with a sealed pedometer (because they were yet to receive the intervention). During visit 4, 12 weeks after visit 1, both groups completed 12-week questionnaires. Also at this visit the immediate group were given a second consultation whilst the delayed group were given an initial physical activity consultation, an individualised incremental 12-week walking programme and a pedometer (just as the immediate group had at visit 2). At visit 5, 23 weeks after visit 1, all participants continued to wear unsealed pedometers (as they had all started the walking programme) and were fitted with an *activ*PAL. All participants completed 24-week questionnaires and were given thank-you packs (consisting of information on local physical activity opportunities and a pedometer) at visit 6, 24 weeks after visit 1. In addition the delayed group were encouraged by the nurse to increase or maintain their walking levels based on whether the individual had met their target step goal over the previous 12 weeks or not. The practice nurse and a research assistant who conducted participant visits were not blinded to group allocation because they were providing the interventions. All participants' information and data were labelled with an identification number. All other researchers were blinded to group allocation.

### Outcome measures

#### i) Physical activity

Daily physical activity was assessed using step counts measured by a sealed pedometer (NL-1000, New Lifestyles Inc, Lee's Summit, Missouri, USA). The NL-1000 records step count and minutes in moderate-to-vigorous intensity activity for the previous 7 days. Participants were instructed to wear the pedometer at all times apart from when washing and sleeping. The NL-1000 threshold for moderate-to-vigorous intensity was set at Level 3, corresponding to walking activity above 2.9 METs [55].

#### ii) Activity patterns

The *activ*PAL monitor (PAL technologies Ltd, Glasgow, Scotland) was used to assess activity in more detail. The *activ*PAL is a small (5 × 3.5 × 0.7 cm), light (20 g) unit which is attached to the anterior surface of the thigh, approximately midway between the inguinal crease and the proximal border of the patella. The monitor measures many variables including total time and percentage of time spent seated/lying, standing and walking, the duration of these events, number of steps per day and during each walking period and the cadence of walking periods. A detailed breakdown of the patterns of daily activity is given by the *activ*PAL as events and periods of time spent in particular postures are time stamped. Data were recorded in 15-second epochs. The *activ*PAL has a battery life of around 9 days and was worn continuously during this period (providing 8 full days of data). The monitor has previously been validated for use with an older population [56].

Physical activity and patterns of physical activity were measured at baseline, between 11 and 12 weeks and between 23 and 24 weeks in both groups over a period of 7-12 days (figure 1). At baseline pedometers were sealed using tape for both groups and at 11 weeks for the delayed group so that participants were not aware of their step counts. After the initial consultation (visit 2 for the immediate group and visit 4 for the delayed group) participants were given an unsealed pedometer. Pedometer and *activ*PAL data collected between visits 1 and 2 gave a baseline reading of physical activity participation for both groups. Between visits 3 and 4 for the immediate group and between visits 5 and 6 for the delayed group, pedometer and *activ*PAL data gave a measure of physical activity participation 12 weeks after the initial consultation. When physical activity was assessed between visits 5 and 6 for the immediate group this gave a measure of participation 12 weeks after the second consultation (i.e. 24 weeks after the initial consultation). Physical activity behaviour was also assessed subjectively using the Stage of Change question [48] at baseline and again at 24 weeks in both groups.

#### ii) Well-being

To determine the efficacy of physical activity interventions for older adults it is important to assess the effects of interventions on the specific areas of health that are important to older adults (e.g. functional capacity, well-being and social interaction). These areas were assessed using the following self-report questionnaires: the Positive and Negative Affect Schedule (PANAS) to assess mood [57]; the Short-Form (36) Health Survey version 2 (SF-36v2) questionnaire which measures quality of life [58]; the Perceived Motor-Efficacy Scale for Older Adults (PMES-OA) to assess functional ability [59]; and the UCLA Loneliness Scale (version 3) [60] as a measure of emotional and social loneliness. Questionnaires were completed by both groups at visits 1, 4 and 6. In addition to these questionnaires, at visit 1 participants completed a demographic questionnaire.

#### iii) Qualitative study

To gain an insight into participants' experiences of the walking intervention, two focus groups were conducted; one with the immediate group, the other with the delayed group (on completion of the 12-week programme). Focus groups are an established method for accessing personal experiences and for facilitating more in-depth understandings of participants' views [61]. All focus groups were facilitated by two members of the research team. Each group consisted of seven to nine participants and lasted for approximately an hour. Questions posed examined both positive and negative experiences. Specifically, the focus group schedule explored perceived benefits of increased walking, views on the pedometer, the physical activity consultation and on-going support, problems encountered, future recommendations and reflections on participation. Focus groups were audio-recorded, transcribed verbatim and thematically analysed (see below). These data, when combined with demographic and efficacy data, will provide insight into the mediators and moderators of change in walking behaviour. In addition, semi-structured interviews were conducted with key members of the research team, in order to explore their experiences of implementing the intervention. The interview schedule covered key questions about the feasibility of implementing the intervention into current care and other settings (including acceptability of intervention training to current general practice staff) and ways of improving the intervention for future delivery. The qualitative evaluation of the intervention supplements the experimental phase by providing context-rich, in-depth information on participants' experiences and provides an insight into the underlying processes that determine the feasibility of the intervention, for instance, what worked, what did not work, the perceived impact and why and how the intervention could be improved for future delivery.

### Setting

Visits were conducted either at a general practice in the West End of Glasgow or in the Glasgow Clinical Research Facility at the Western Infirmary, Glasgow (http://www.glasgowcrf.org.uk). Focus groups were conducted in a university building or a suitable community venue.

### Data management

Quantitative data were entered into a Microsoft Excel (Microsoft Corp., Redmond, WA, USA) database and stored on a secure network drive. All questionnaire data were double-entered and checked by a member of the research team. Qualitative data were audio-recorded, transcribed verbatim and anonymised. Hard copies of questionnaire data and the focus group and interview transcripts were kept in a secure filing cabinet.

### Data analysis

Data collection was completed in December 2010. Inferences about the effect of the intervention will be drawn from comparisons between groups in outcome measurements at baseline and at 12 weeks. Follow-up data at 24 weeks will be used in the immediate group to assess whether any benefits are maintained; in the delayed group follow-up data will provide a secondary assessment of the impact of the intervention. Data will be analysed on an intention to treat basis, that is according to the randomisation rather than whether or not the intervention was received or completed. In the first instance, missing data will not be imputed, though the sensitivity of the main findings will be assessed under alternative assumptions regarding missing values (such as last observation carried forward). Similarly, outlying values (e.g. very low step counts) will not be excluded from initial analyses, though analyses will be repeated following the exclusion of outcome values that fall outside plausible ranges. Qualitative data will be thematically analysed, which involves coding participants' speech into categories that summarise and systemise the content of the data [62]. NVivo 8 software (QSR International, Melbourne, Australia) will be used to aid coding and data retrieval.

## Discussion

This paper describes the WEW65+ study rationale and design, including details of the intervention, the outcome measures and recruitment process. WEW65+ will provide information on the delivery of a walking intervention through a primary care setting specifically via a general practice (feasibility element of the study) as well as the efficacy of the intervention in men and women aged ≥65 years (pilot element of the study). The study will address several evidence gaps: a) exploration of the efficacy of an intervention to increase walking participation using an objective measure of physical activity in a randomised controlled trial with an older adult population in the UK; b) determination of the achievability of the physical activity recommendation for older adults; c) examination of the feasibility of recruiting older adults via a general practice; and d) assessment of the delivery of the walking intervention in a primary care setting (general practice) by a practice nurse.

### Strengths

Findings from this study will determine if the intervention is efficacious and feasible and thus if it should then be tested in a larger trial of effectiveness, in real-world conditions (based on current guidance on evaluation of complex interventions [41]). The framework objectives for phase II pilot and feasibility studies are as follows: to have confidence that the intervention can be delivered as planned; to be able to make 'safe assumptions about effect sizes and variability;' and to be confident of recruitment and retention rates. The Consolidated Standards for Reporting Trials (CONSORT), guidelines were designed to improve the design and reporting of randomised controlled trials [63]. By following these guidelines the process of putting research into practice can be enhanced [49]. Principles from the CONSORT guidelines were used to strengthen the design of this study.

#### Objective measures of physical activity

In this study the intervention is centred round the current physical activity recommendation for older adults and will inform whether this recommendation is achievable. Physical activity participation is measured using more than one objective measure in this study which will allow the comparison of step counts and other physical activity output (e.g., step rate and walking intensity) from the devices and provides a back-up should one device fail or work incorrectly. In addition to measuring physical activity, the *activ*PAL measures sedentary time and these data will help to determine whether a recommendation for older adults on sedentary behaviour is required, and whether a walking intervention has a supplementary effect on sedentary behaviour.

#### Well-being assessment

The collection of well-being and functional ability data (mood, quality of life, perceived motor-efficacy and loneliness) are strengths of this study as these areas of health are extremely important in older people [2,12]. Well-being data will also help facilitate cost-effectiveness analysis of the intervention in the future.

#### Mixed methods approach and lessons from previous research

This study combines quantitative and qualitative elements in order to address the research questions as comprehensively as possible.

#### Multi-disciplinary team

The research team for this project consisted of 13 members from various disciplinary backgrounds (including physical activity researchers, psychologists, a sociologist, a measurement specialist, a general practitioner and a statistician). Steering group meetings were held monthly to drive the development of the study.

### Challenges

Several challenges should be considered when designing an intervention to be delivered via a general practice setting for older adults.

#### Delivering a walking intervention via a general practice

The study was based entirely in primary care (including recruitment, intervention delivery and follow-up). It was recognised that time scheduling in general practices is tight and thus we attempted to make the intervention not too time intensive. The duration of each study visit is between 5 and 60 minutes (see Figure 1 for visit time length). In this study there were 6 study visits in total. However three study visits (visits 1, 3 and 5) were for the measurement of physical activity. These visits would not be required if the walking intervention was to be implemented in general practices without the research components included in the WEW65+ study.

#### Targeting older adults

The trial recruited both men and women aged ≥65 years. Population data for Scotland highlight the alarmingly high proportion of older men and women that are inactive. Also, population data show that walking declines steadily with age in both men and women and that this decline is more marked and occurs earlier in men [3]. Thus efforts are required to encourage both older men and women to increase their walking levels by developing interventions that target and are acceptable for both genders.

#### Screening for participation in physical activity

Little is known about how best to screen for physical activity participation and who is able to do this. In this study the general practitioner was the only team member granted full ethical access to medical records and who could therefore complete screening for eligibility into the study. An outcome of this study will be the development of a list of physical activity contraindications that are specific to an older adult population which can be used to aid physical activity screening in the future and could be used by other primary care staff, removing the requirement of a general practitioner for eligibility screening.

#### Screening out those who are already active

Defining who is 'already active' and 'insufficiently active' and the cut-off points for these using both objective and subjective measures of physical activity is a major challenge in physical activity intervention research. In an attempt to ensure that insufficiently active individuals were included in this study and already active individuals were excluded from participation the protocol had two physical activity level screening phases (verbal/written screening with a researcher and objective screening based on pedometer step counts). It is possible that during the initial screening phase individuals will over or under report physical activity. Objective physical activity readings can be affected by 'reactivity' [64] (when an individual changes their normal physical activity pattern because they are aware that their activity levels are being assessed) or wear compliance (e.g. forgetting to wear the activity monitor or not wearing it in the correct position). The use of self-report measures of physical activity, reactivity and wear compliance could adversely affect recruitment into the study. Individuals who are already regularly active may be incorrectly recruited (if for example they under-report the amount of activity they do or forget to wear the pedometer for all waking hours). Conversely, reactivity can lead to relatively inactive individuals incorrectly being excluded from study participation (if they walk more than usual during the monitoring period).

### Limitations

#### Generalisability

Participants were recruited via a single general practice thus the feasibility findings may have limited generalisability.

#### Physiological health and cost-effectiveness outcomes

Another limitation of this study is the lack of physiological health outcomes due to funding constraints. However, the health benefits of walking are already well-documented, whereas research is lacking in the area of determining how best to support people to walk regularly. A cost-effectiveness analysis will not be conducted in this study but the gathering of well-being data will aid such analysis in the future.

#### Comparison group

As mentioned earlier the CONSORT guidelines were used to guide the design of this study [63]. This study did not have a control group over the full study period as it was viewed as unethical to withhold the walking intervention and consequently the possible health benefits that could occur from increasing physical activity participation. Thus we opted for a 'delayed' group rather than a true control group for the entire length of the study.

## Conclusions

The feasibility elements of WEW65+ will inform research in the area of physical activity intervention delivery through a primary care setting via a general practice. The pilot component of this study examines efficacy of a walking intervention (already shown to be effective in younger adults [17]) in an older adult population in Glasgow, by measuring pedometer step counts as well as secondary measures including well-being. Useful information as to why individuals did not wish to participate in this study and the reasons for exclusion from the study during the screening process will aid the design of future physical activity interventions in older adults.

## Competing interests

The authors declare that they have no competing interests. Professor Malcolm Granat is a co inventor of the activity monitor and a director of PAL Technologies Ltd, Glasgow UK. Professor Granat had no involvement in the data collection. There was no financial contribution to this work from PAL Technologies Ltd.

## Authors' contributions

FM CF and NM led the drafting and editing of the manuscript. KB provided primary care expertise to make the protocol feasible in a general practice setting. CF, MHG, PMG, MG, HM, AM, DR, RS, DS and NM were involved in the original application and design of the study. All authors read and approved the final manuscript.

## Pre-publication history

The pre-publication history for this paper can be accessed here:

http://www.biomedcentral.com/1471-2458/11/120/prepub

## References

[B1] Department of Health On the state of public health: Annual report of the Chief Medical Officer 2009 2010 Department of Health: London

[B2] Nelson ME Rejeski WJ Blair SN Duncan PW Judge JO King AC Macera CA Castaneda-Sceppa C Physical activity and public health in older adults. Recommendation from the American College of Sports Medicine and the American Heart Association Circulation 2007 116 9 1094 1105 10.1161/CIRCULATIONAHA.107.185650 17671236

[B3] Executive Scottish The Scottish Health Survey 2009 2010 Crown Copyright: Edinburgh

[B4] Executive Scottishlet's make Scotland more active: A strategy for physical activity 2003 Crown Copyright: Edinburgh

[B5] NHS Health Scotland Five year review of 'Let's Make Scotland More Active' - A strategy for physical activity 2009 NHS Health Scotland: Edinburgh

[B6] The Futures Project: Trend analysis papers http://www.scotland.gov.uk/Publications/2006/05/22134120/0

[B7] Strawbridge WJ Kaplan GA Camacho T Cohen RD The dynamics of disability and functional change in an elderly cohort: Results from the Alameda county study Journal of the American Geriatrics Society 1992 40 8 799 806 1386090 10.1111/j.1532-5415.1992.tb01852.x

[B8] Sonn U Longitudinal studies of dependence in daily life activities among elderly persons Scandinavian Journal of Rehabilitation Medicine Supplement 1996 34 1 35 8701230

[B9] Bennett KM A longitudinal study of wellbeing in widowed women International Journal of Geriatric Psychiatry 1997 12 1 61 66 10.1002/(SICI)1099-1166(199701)12:1<61::AID-GPS465>3.0.CO;2-U 9050425

[B10] Luo L Craik FI Aging and memory: A cognitive approach Canadian Journal of Psychiatry 2008 53 6 346 353 10.1177/070674370805300603 18616854

[B11] Anstey KJ Low L-F Normal cognitive changes in aging Australian Family Physician 2004 33 10 783 787 15532151

[B12] Department of Health At least five a week: Evidence on the impact of physical activity and its relationship to health. A report from the Chief Medical Officer 2004 London 128 128

[B13] NHS Health Scotland Energising lives: A guide to promoting physical activity in primary care 2008 NHS Health Scotland: Edinburgh

[B14] O'Donovan G Blazevich AJ Boreham C Cooper AR Crank H Ekelund U Fox KR Gately P Giles-Corti B Gill JMR The ABC of physical activity for health: A consensus statement from the British Association of Sport and Exercise Sciences Journal of Sports Sciences 2010 28 6 573 591 20401789 10.1080/02640411003671212

[B15] Morris JN Hardman AE Walking to health Sports Medicine 1997 23 5 306 333 10.2165/00007256-199723050-00004 9181668

[B16] Murphy MH Nevill AM Murtagh EM Holder RL The effect of walking on fitness, fatness and resting blood pressure: A meta-analysis of randomised, controlled trials Preventive Medicine 2007 44 5 377 385 10.1016/j.ypmed.2006.12.008 17275896

[B17] Baker G Gray S Wright A Fitzsimons C Nimmo M Lowry R Mutrie N the Scottish Physical Activity Research Collaboration The effect of a pedometer-based community walking intervention "Walking for Wellbeing in the West" on physical activity levels and health outcomes: A 12-week randomized controlled trial International Journal of Behavioral Nutrition and Physical Activity 2008 5 5 44 49 18775062 10.1186/1479-5868-5-44 PMC2546435

[B18] Ogilvie D Foster CE Rothnie H Cavill N Hamilton V Fitzsimons CF Mutrie N on behalf of Scottish Physical Activity Research Collaboration Interventions to promote walking: Systematic review British Medical Journal 2007 334 7605 1204 1207 10.1136/bmj.39198.722720.BE 17540909 PMC1889976

[B19] Scottish Executive Improving health in Scotland - The challenge 2003 Crown Copyright

[B20] Katzmarzyk PT Church TS Craig CL Bouchard C Sitting time and mortality from all causes, cardiovascular disease, and cancer Medicine & Science in Sports & Exercise 2009 41 5 998 1005 10.1249/MSS.0b013e3181930355 19346988

[B21] Sugiyama T Healy G Dunstan D Salmon J Owen N Joint associations of multiple leisure-time sedentary behaviours and physical activity with obesity in Australian adults International Journal of Behavioral Nutrition and Physical Activity 2008 5 35 10.1186/1479-5868-5-35 18590570 PMC2459202

[B22] Grant PM Granat MH Thow MK Maclaren WM Analyzing free-living physical activity of older adults in different environments using body-worn activity monitors Journal of Aging and Physical Activity 2010 18 2 171 184 20440029 10.1123/japa.18.2.171

[B23] Fitzsimons C Baker G Wright A Nimmo M Ward Thompson C Lowry R Millington C Shaw R Fenwick E Ogilvie D The 'Walking for Wellbeing in the West' randomised controlled trial of a pedometer-based walking programme in combination with physical activity consultation with 12 month follow-up: Rationale and study design BMC Public Health 2008 8 259 10.1186/1471-2458-8-259 18655723 PMC2518560

[B24] Kirk A Barnett J Mutrie N Physical activity consultation for people with Type 2 diabetes: Evidence and guidelines Diabetic Medicine 2007 24 8 809 816 10.1111/j.1464-5491.2007.02190.x 17650156

[B25] Abraham C Michie S A taxonomy of behavior change techniques used in interventions Health Psychology 2008 27 3 379 387 10.1037/0278-6133.27.3.379 18624603

[B26] Wood R Bain MRS The health and well-being of older people in Scotland: Insights from national data 2001 Edinburgh: Information & Statistics Division

[B27] Information & Statistics Division General practice - Practice team information 2010 http://www.isdscotland.org/isd/1044.html

[B28] Eakin EG Glasgow RE Riley KM Review of primary care-based physical activity intervention studies: Effectiveness and implications for practice and future research Journal of Family Practice 2000 49 2 158 168 10718694

[B29] Laurant M Reeves D Hermens R Braspenning J Grol R Sibbald B Substitution of doctors by nurses in primary care Cochrane Database of Systematic Reviews 2004 4 CD001271 10.1002/14651858.CD001271.pub2 15846614

[B30] National Institute for Health and Clinical Excellence Four commonly used methods to increase physical activity: Brief interventions in primary care, exercise referral schemes, pedometers and community-based exercise programmes for walking and cycling 2006 London: National Institute for Health and Clinical Excellence

[B31] Swinburn BA Walter LG Arrol B Tilyard MW Russell DG The green prescription study: A randomized controlled trial of written exercise advice in general practice American Journal of Public Health 1998 88 2 288 291 10.2105/AJPH.88.2.288 9491025 PMC1508188

[B32] Bull FC Jamrozik K Advice on exercise from a family physician can help sedentary patients to become active American Journal of Preventive Medicine 1998 15 2 85 94 10.1016/S0749-3797(98)00040-3 9713663

[B33] Nanchahal K Townsend J Letley L Haslam D Wellings K Haines A Weight-management interventions in primary care: A pilot randomised controlled trial British Journal of General Practice 2009 59 562 e157 166 10.3399/bjgp09X420617 19401009 PMC2673183

[B34] Hardcastle S Taylor A Bailey M Castle R A randomised controlled trial on the effectiveness of a primary health care based counselling intervention on physical activity, diet and CHD risk factors Patient Education and Counseling 2008 70 1 31 39 10.1016/j.pec.2007.09.014 17997263

[B35] Greaves CJ Middlebrooke A O'Loughlin L Holland S Piper J Steele A Gale T Hammerton F Daly M Motivational interviewing for modifying diabetes risk: A randomised controlled trial British Journal of General Practice 2008 58 553 535 540 10.3399/bjgp08X319648 18682011 PMC2566518

[B36] Kinmonth AL Wareham NJ Hardeman W Sutton S Prevost AT Fanshawe T Williams KM Ekelund U Spiegelhalter D Griffin SJ Efficacy of a theory-based behavioural intervention to increase physical activity in an at-risk group in primary care (ProActive UK): A randomised trial Lancet 2008 371 9606 41 48 10.1016/S0140-6736(08)60070-7 18177774

[B37] McKay J Wright A Lowry R Steele K Ryde G Mutrie N Walking on prescription: The utility of a pedometer pack for increasing physical activity in primary care Patient Education Counseling 2009 76 1 71 76 10.1016/j.pec.2008.11.004 19097843

[B38] Yates T Davies M Gorely T Bull F Khunti K Effectiveness of a pragmatic education program designed to promote walking activity in individuals with impaired glucose tolerance: A randomized controlled trial Diabetes Care 2009 32 8 1404 1410 10.2337/dc09-0130 19602539 PMC2713638

[B39] Sugden JA Sniehotta FF Donnan PT Boyle P Johnston DW McMurdo ME The feasibility of using pedometers and brief advice to increase activity in sedentary older women - A pilot study BMC Health Services Research 2008 8 169 18691392 10.1186/1472-6963-8-169 PMC2527003

[B40] Bull FC Milton KE A process evaluation of a "physical activity pathway" in the primary care setting BMC Public Health 10 463 10.1186/1471-2458-10-463 20696030 PMC2933718

[B41] Craig P Dieppe P Macintyre S Mitchie S Nazareth I Petticrew M Developing and evaluating complex interventions: The new Medical Research Council guidance British Medical Journal 2008 337 979 983 10.1136/bmj.a1655 PMC2769032 18824488

[B42] National Institute for Health Research Evaluation, Trials and Studies Coordinating Centre Glossary http://www.netscc.ac.uk/glossary/

[B43] Glasgow RE Vogt TM Boles SM Evaluating the public health impact of health promotion interventions: The RE-AIM framework American Journal of Public Health 1999 89 9 1322 1327 10.2105/AJPH.89.9.1322 10474547 PMC1508772

[B44] Rowe D Kemble C Robinson T Mahar M Daily walking in older adults: Day-to-day variability and criterion-referenced validity of total daily step counts Journal of Physical Activity and Health 2007 4 4 434 336 18209234

[B45] Stel VS Smit JH Pluijm SMF Visser M Deeg DJH Lips P Comparison of the LASA Physical Activity Questionnaire with a 7-day diary and pedometer Journal of Clinical Epidemiology 2004 57 3 252 258 10.1016/j.jclinepi.2003.07.008 15066685

[B46] Cohen J Statistical power analysis for the social sciences 1988 2 New York: Routledge Academic

[B47] Greig CA Young A Skelton DA Pippet E Butler FM Mahmud SM Exercise studies with elderly volunteers Age and Ageing 1994 23 3 185 189 10.1093/ageing/23.3.185 8085501

[B48] Marcus BH Simkin LR The Transtheoretical Model: Applications to exercise behaviour Medicine and Science in Sports and Exercise 1994 26 11 1400 1404 7837962

[B49] Glasziou P Chalmers I Altman DD Bastian H Boutron I Brice A Jamtvedt G Farmer A Ghersi D Groves T Taking healthcare interventions from trial to practice British Medical Journal 2010 341 c3852 10.1136/bmj.c3852 20709714

[B50] Kirk A Mutrie N MacIntyre P Fisher M Effects of a 12-month physical activity counselling intervention on glycaemic control and on the status of cardiovascular risk factors in people with Type 2 diabetes Diabetologia 2004 47 5 821 832 10.1007/s00125-004-1396-5 15138687

[B51] Hughes A Mutrie N Exercise consultation improves exercise adherence in phase IV cardiac rehabilitation Journal of Cardiopulmonary Rehabilitation 2002 22 6 421 425 10.1097/00008483-200211000-00007 12464830

[B52] Beets MW Agiovlasitis S Fahs CA Ranadive SM Fernhall B Adjusting step count recommendations for anthropometric variations in leg length Journal of Science and Medicine in Sport 2010 13 5 509 512 10.1016/j.jsams.2009.11.002 20096631

[B53] Rowe D Welk G Heil D Kemble C Calabro M Camenisch K Mahar M Stride rate guidelines for moderate intensity walking Medicine and Science in Sport and Exercise 2010 http://journals.lww.com/acsm-msse/Abstract/publishahead/Stride_Rate_Recommendations_for_Moderate_Intensity.99166.aspx electronic version available ahead of print at 10.1249/MSS.0b013e3181e9d99a 20543754

[B54] Tudor-Locke C Sisson SB Collova T Lee SM Swan PD Pedometer-determined step count guidelines for classifying walking intensity in a young ostensibly healthy population Canadian Journal of Applied Physiology 2005 30 6 666 676 16485518 10.1139/h05-147

[B55] McClain JJ Sisson SB Washington TL Tracy L Craig CL Tudor-Locke C Comparison of Kenz Lifecorder EX and ActiGraph accelerometers in 10-yr-old children Medicine and Science in Sports and Exercise 2007 39 4 630 638 10.1249/mss.0b013e3180313056 17414800

[B56] Grant PM Dall PM Mitchell SL Granat MH Activity-monitor accuracy in measuring step number and cadence in community-dwelling older adults Journal of Aging and Physical Activity 2008 16 2 201 214 18483442 10.1123/japa.16.2.201

[B57] Watson D Clark LA Tellegen A Development and validation of brief measures of positive and negative affect: The PANAS Scales Journal of Personality and Social Psychology 1988 54 6 1063 1070 10.1037/0022-3514.54.6.1063 3397865

[B58] Jenkinson C Stewart-Brown S Petersen S Paice C Assessment of the SF-36 version 2 in the United Kingdom Journal of Epidemiology & Community Health 1999 53 1 46 50 10.1136/jech.53.1.46 PMC1756775 10326053

[B59] Potter LM Grealy MA O'Connor RC Healthy ageing, perceived motor-efficacy, and performance on cognitively demanding action tasks British Journal of Psychology 2009 100 Pt 1 49 70 10.1348/000712608X304478 18447971

[B60] Russell DW UCLA Loneliness Scale (Version 3): Reliability, validity, and factor structure Journal of Personality Assessment 1996 66 1 20 40 10.1207/s15327752jpa6601_2 8576833

[B61] Kitzinger J Introducing focus groups in qualitative research British Medical Journal 1995 311 7000 299 302 7633241 10.1136/bmj.311.7000.299 PMC2550365

[B62] Braun V Clarke V Using thematic analysis in psychology Qualitative research in psychology 2006 3 2 77 101 10.1191/1478088706qp063oa

[B63] Schulz KF Altman DG Moher D CONSORT 2010 statement: Updated guidelines for reporting parallel group randomised trials British Medical Journal 2010 340 c332 10.1136/bmj.c332 20332509 PMC2844940

[B64] Welk GJ Corbin CB Dale D Measurement issues in the assessment of physical activity in children Research Quarterly for Exercise and Sport 2000 71 Suppl 2 S59 S73 10925827

